# Homeostasis of metabolites in *Escherichia coli* on transition from anaerobic to aerobic conditions and the transient secretion of pyruvate

**DOI:** 10.1098/rsos.160187

**Published:** 2016-08-24

**Authors:** Nur Adeela Yasid, Matthew D. Rolfe, Jeffrey Green, Mike P. Williamson

**Affiliations:** Department of Molecular Biology and Biotechnology, University of Sheffield, Firth Court, Western Bank, Sheffield S10 2TN, UK

**Keywords:** *Escherichia coli*, metabolomics, metabolon, glycolysis, nuclear magnetic resonance

## Abstract

We have developed a method for rapid quenching of samples taken from chemostat cultures of *Escherichia coli* that gives reproducible and reliable measurements of extracellular and intracellular metabolites by ^1^H NMR and have applied it to study the major central metabolites during the transition from anaerobic to aerobic growth. Almost all metabolites showed a gradual change after perturbation with air, consistent with immediate inhibition of pyruvate formate-lyase, dilution of overflow metabolites and induction of aerobic enzymes. Surprisingly, although pyruvate showed almost no change in intracellular concentration, the extracellular concentration transiently increased. The absence of intracellular accumulation of pyruvate suggested that one or more glycolytic enzymes might relocate to the cell membrane. To test this hypothesis, chromosomal pyruvate kinase (*pykF*) was modified to express either PykF-green fluorescent protein or PykF-FLAG fusion proteins. Measurements showed that PykF-FLAG relocates to the cell membrane within 5 min of aeration and then slowly returns to the cytoplasm, suggesting that on aeration, PykF associates with the membrane to facilitate secretion of pyruvate to maintain constant intracellular levels.

## 1. Introduction

*Escherichia coli* is a metabolically versatile bacterium. In the presence of oxygen, it grows by aerobic respiration. When growing on glucose, it can completely oxidize it via glycolysis to pyruvate, which is taken on to the citric acid cycle and electron transport chain by the pyruvate dehydrogenase complex (PDHC). This mode of growth is by far the most energy efficient [1]. In the absence of oxygen, it can grow by anaerobic respiration, if there is a terminal electron acceptor available, such as the nitrate ion. If there is no such electron acceptor, then it can grow by fermentation. This is the least energy efficient of the three modes, and in order to achieve redox balance it produces overflow metabolites (acetate, ethanol, formate, lactate and succinate) which it secretes into the growth medium. Under fermentative conditions glycolysis proceeds to pyruvate in the same way as for aerobic metabolism, but thereafter pyruvate is metabolized by pyruvate formate-lyase (PFL), because PDHC has very low activity [2]. Whereas PDHC converts pyruvate into acetyl CoA and CO_2_, with concomitant reduction of NAD^+^ to NADH, PFL produces acetyl CoA and formate only. The acetyl CoA produced anaerobically is converted into coenzyme A and acetate via acetylphosphate, allowing energy conservation by substrate-level phosphorylation of adenosine diphosphate (ADP), and to ethanol in a process that reoxidizes the NADH generated during glycolysis. The metabolic pathway for fermentative growth is thus radically different from aerobic growth, from pyruvate onwards. The transition from anaerobic to aerobic growth is important, because *E. coli* grows both anaerobically in the intestine and aerobically when excreted. Its ability to switch rapidly from one growth mode to another is crucial for its success.

The steady-state growth of *E. coli* is well characterized. Useful results have been obtained using chemostats [3,4], which are growth vessels that make use of a constant influx of growth medium, balanced by a constant outflow of cell suspension, and thus maintain a steady state, with a constant cell density and specific growth rate, controlled by the dilution rate of the medium, as well as by the composition of the medium, pH, temperature, and gas flow, which can all be continuously monitored. Samples can be withdrawn at any time. Here, a chemostat was used to produce reproducible steady-state anaerobic conditions. The transition to aerobic respiratory growth was started by introducing oxygen into the gas supply, and samples were taken at various time points thereafter.

Transcriptional changes have been studied in this system [5]. Within 5 min of the introduction of oxygen, the abundances of transcripts associated with anaerobic metabolism were decreased, while those of aerobic metabolism were increased. A good correspondence between transcript level and protein level was observed [6]. The transition to microaerobic growth has a similar behaviour [6,7]. In both cases, there is rapid inactivation of PFL [8] (thought to be caused by oxygenolysis of this free radical enzyme [9]) and a slower induction of PDHC. Concentrations of extracellular metabolites were measured, and fitted the expected pattern [6]. Under steady-state anaerobic conditions in glucose-limited (21.9 mM) cultures, the cells secreted formate (35 mM), acetate (19 mM), ethanol (10 mM), succinate (5 mM) and lactate (0.1 mM). After introduction of oxygen, the concentrations of most extracellular metabolites decreased at a rate corresponding to the dilution rate, implying that secretion of overmetabolites stopped immediately. However, the concentration of acetate decreased more slowly, while fumarate increased during the transition. An interesting feature was a transient increase in extracellular pyruvate, which rose from undetectable under anaerobic conditions to 1 mM at 1 h and then decreased, with the maximum excretion rate being at around 10 min. The change was interpreted as being consistent with a large transient increase in intracellular pyruvate, followed by secretion into the medium [6]. Gradually, the intracellular activity of PDHC increased, and after a few hours pyruvate was no longer detectable in the medium.

Here, we measured metabolite changes during the transition from anaerobic to aerobic growth. The concentrations of most extracellular metabolites gradually decreased. This was consistent with the inactivation of PFL and initiation of aerobic metabolism. However, as previously reported, a transient increase in extracellular pyruvate was observed but we show that this was not accompanied by an increase in intracellular pyruvate concentration. We therefore hypothesized that pyruvate was being excreted into the medium without accumulating in the cytoplasm, which would require relocation of glycolytic enzymes to the cell membrane. This was tested by making a replacement copy of chromosomal pyruvate kinase (PykF), tagged either with green fluorescent protein (GFP) or with a FLAG tag, in order to visualize the location of PykF in the bacterial cells by fluorescence microscopy or by electron microscopy following washing with a nanogold particle-tagged secondary antibody. The results suggest that a significant fraction of the cytoplasmic PykF relocated to the cell membrane on introduction of oxygen, possibly as part of a glycolytic metabolon regulated by aerobic stress.

## 2. Results

### 2.1. Nuclear magnetic resonance-based metabolite assay

Intracellular metabolite concentrations can change extremely rapidly: it is estimated that glycolytic metabolites in yeast can alter by as much as a factor of two within 1 s [10]. It is therefore vital that cell metabolism should be quenched very rapidly after sampling, but without forming intracellular ice crystals, which damage the membrane and lead to leakage of cell contents. The most popular method for rapid quenching is to mix the cells with cold methanol [10,11], methanol/chloroform [12] or methanol/glycerol [13], after which the cells can be separated from the supernatant by centrifugation. However, this tends to make the cell membrane leaky, and there is significant loss of metabolites [14]. It has therefore been suggested to use ethanol and saline instead of methanol, which was shown to induce much less leakage, particularly when the saline concentration is adjusted to match the intracellular osmotic strength [14–16]. Our modified method, therefore, consists of mixing bacterial culture samples (at 37°C) with an equal volume of ethanol/saline at −35°C. This cools the cells within about 1 s to about 1°C. Cells were further cooled to −5°C within about 1 min, after which they were centrifuged at −11°C to separate cells from supernatant. Because the volume of supernatant is very much larger than the volume of cells, this is the crucial separation. After this, as long as the cells are kept cold, further metabolism or leakage is much less critical. A number of methods have been used for extraction of metabolites, of which the most popular method has been extraction using perchloric acid [17]. However, this leads to degradation of some metabolites, and an alternative methodology using methanol was preferred [18]. The cell pellet was therefore cooled to −80°C in methanol and subjected to at least three freeze–thaw cycles followed by centrifugation and lyophilization of the cell supernatant, which was redissolved in D_2_O and measured using ^1^H nuclear magnetic resonance (NMR). Lyophilization removes volatile metabolites such as ethanol, but is worthwhile because replacement of H_2_O by D_2_O leads to a much flatter baseline in NMR and, therefore, improved quantitation. A good test of rapid quenching is to grow cells aerobically; any metabolism subsequent to sampling results in production of succinate and acetate due to exhaustion of oxygen and switching to fermentative metabolism. No succinate or acetate were detected, confirming that quenching halts metabolism very rapidly. A good test for cell leakage is to look for glutamate in cell supernatant. Glutamate is the most abundant metabolite in *E. coli* [19] and is not secreted into the medium. No glutamate could be detected in the supernatant of cells quenched using ethanol/saline (electronic supplementary material, figure S1), implying that there is no more than 5% leakage of intracellular metabolites into the supernatant as a result of the quenching. This level of leakage is too small to affect our results in any important way. A more accurate method for quantitation of intracellular and extracellular metabolites would be a differential method [20]. However, the low level of leakage in our system makes such a method unnecessary.

The reproducibility of the measurement was tested (electronic supplementary material, table S1). Most factors caused little error, and extracellular metabolites could be measured within an error of 2%. The error in measurement of intracellular metabolite concentrations was much larger, and came from two main sources: cell handling during quenching; and the conversion from relative concentration to absolute concentration. After optimization (described in Material and methods), the handling error was estimated at 14%. The conversion to absolute concentration was done in several ways. The most reliable was by using a simple estimation of the average intracellular volume of a single *E. coli* cell as 0.5 fl [21–24], multiplied by the number of cells (determined from the optical density). This is clearly a crude average, and any error or sample-to-sample variation in this figure will lead to a rescaling of all derived concentrations. However, as rescaling affects absolute concentrations only, the relative concentrations described here are subject only to the handling errors.

### 2.2. Extracellular metabolites

*Escherichia coli* cells were grown in chemostat cultures with a dilution rate of 0.2 h^−1^. The growth medium was chosen to ensure that cell growth was glucose limited. This was confirmed by NMR spectra of culture supernatants, which never showed detectable amounts of glucose. As expected, the cultures contained almost no extracellular metabolites under steady-state aerobic conditions, because glucose is completely oxidized (electronic supplementary material, figure S2*a*). By comparison, steady-state anaerobic cultures contained large signals from formate, ethanol, succinate and acetate (electronic supplementary material, figure S2*b*), corresponding to concentrations of 42, 11, 4 and 17 mM, respectively. These are all well-known extracellular metabolites [6]. A useful check on the results comes from the carbon balance (C number). Under anaerobic conditions, the concentration of carbon entering the system is 25.1 mmol C h^−1^ (24 from glucose, i.e. 20 mM × 1 l × 0.2 h^−1^ × 6 carbons, plus 1.1 from CO_2_). The carbon output is 8.4 (formate) + 6.8 (acetate) + 3.0 (succinate) + 4.2 (ethanol) + 0.04 (lactate) + 3.0 (biomass, assuming 44.3% of cell dry weight is carbon [20]) = 25.44, giving a carbon balance of 101.4%. Similar results were obtained in our previous study [6].

**Figure 1.**
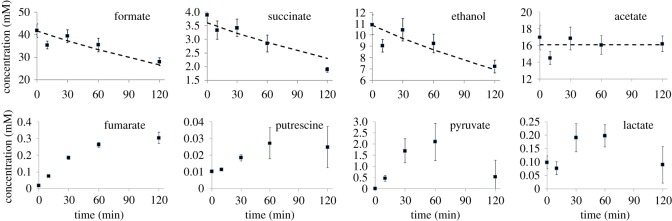
Concentrations of extracellular metabolites following introduction of oxygen to anaerobic culture. Errors are standard deviations (*n *= 3 independent cultures). For formate, succinate and ethanol, the dashed lines show a best fit to a decay of 0.2 h^−1^; for acetate, the dashed line is constant.

Upon perturbation of the anaerobic steady state by the introduction of air, extracellular metabolite concentrations changed (figure 1). It was expected that anaerobic metabolism would cease almost immediately after introduction of oxygen. Accordingly, cell mass increased after the introduction of air with no discernible pause (dry cell mass = 0.40, 0.41, 0.41, 0.46 and 0.64 mg ml^−1^ at 0, 10, 30, 60 and 120 min, respectively), consistent with the greater efficiency of aerobic metabolism. Moreover, the production of the key overmetabolites ethanol, formate and succinate decreased at a rate statistically indistinguishable from the ‘wash-out’ rate of 0.2 h^−1^ (by *t*-test). The apparent shutdown of formate production is consistent with the inactivation of the oxygen-sensitive enzyme, PFL, which converts pyruvate to acetyl CoA and formate under anaerobic conditions [8,9]. By contrast, the concentration of acetate remained unchanged, suggesting that a significant proportion of the acetyl CoA, now generated by the action of the PDHC, was converted to acetate via the phosphotransacetylase/acetate kinase enzymes. This is possible because the presence of an electron acceptor (oxygen) spares the need to produce ethanol in order to achieve redox balance. Alternatively, some acetate might be produced by the action of pyruvate oxidase, a stationary phase peripheral membrane protein [25]. The extracellular concentration of putrescine was very low but increased gradually. The concentration of fumarate rose steadily from very low levels, and the concentrations of pyruvate and lactate rose from very low or undetectable levels, peaked at 1 h and then decayed (figure 1).

The transient rise of extracellular pyruvate is particularly interesting. Under anaerobic conditions, pyruvate is metabolized by PFL, which is inactivated essentially immediately and completely on introduction of oxygen [8]. Under aerobic conditions, pyruvate is metabolized by the PDHC. However, PDHC is inhibited by high NADH/NAD^+^ ratios [26,27], and the NADH/NAD^+^ ratio has been shown to remain high throughout the first hour following introduction of oxygen [5,28]. In agreement with these expectations, the PDHC activity under our conditions over the first 30 min is less than 1 mmol pyruvate consumed h^−1^ g dry cell weight^−1^ [6], equivalent to less than 1.2 mmol C h^−1^ in the culture, where the flux through glycolysis is approximately 22 mmol C h^−1^ (excludes C that enters biomass). Thus, the activity of PDHC is insufficient to handle the large amounts of pyruvate accumulating following inactivation of PFL. The concentration of extracellular pyruvate grows by approximately 12 mmol C h^−1^ over the first 30 min, after which it slows down and then decreases. The concentration of acetate remains constant at 6.8 mmol C h^−1^ and fumarate and lactate increase at 0.6 and 1.6 mmol C h^−1^, respectively. Therefore, our results demonstrate that over the first 30 min almost all of the pyruvate generated by glycolysis is directly excreted from the cell, but that by 2 h most of the pyruvate excreted has been taken up by the cells again. This time scale matches the expected increase of PDHC activity, as a result of a sixfold upregulation of PDHC expression [5] combined with removal of inhibition that results from the high NADH/NAD^+^ ratio present during anaerobic fermentative growth. It therefore appears that the cells excrete pyruvate until the intracellular PDHC activity becomes sufficient to metabolize the pyruvate produced by glycolysis, at which point pyruvate remains within the cells.

### 2.3. Intracellular metabolites

**Figure 2.**
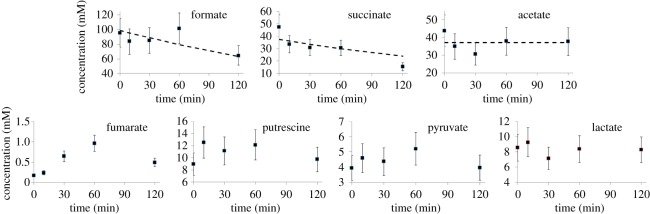
Concentrations of intracellular metabolites following introduction of oxygen to anaerobic culture. Errors are standard deviations (*n *= 3 independent cultures). For formate and succinate, the dashed lines show a best fit to a decay of 0.2 h^−1^; for acetate, the dashed line is constant.

The most striking observation is that intracellular concentrations of all the major metabolites simply change gradually from the steady-state anaerobic concentrations to the steady-state aerobic concentrations, without any kind of a hiatus during the transition (figure 2). Intracellular concentrations of formate and succinate decreased by 0.2 h^−1^, within error (figure 2). This result is as expected from the extracellular measurements and is consistent with immediate cessation of fermentative metabolism on addition of oxygen. We were unable to measure the intracellular concentration of ethanol, as in our method, ethanol is removed by lyophilization. Acetate concentrations remained approximately constant, while fumarate concentrations rose (figure 2), again consistent with extracellular measurements. Concentrations of putrescine, lactate and pyruvate remained approximately constant (figure 2). Genes involved in the degradation of putrescine are induced during the transition from anaerobic to fully aerobic conditions [5]. We also note that Trotter *et al*. [6] saw an increase in extracellular putrescine during the transition, as seen here. Hence, it is entirely plausible that the reported changes to chromosome structure [5] arose from changes to the DNA-bound putrescine pool, liberating free intracellular putrescine and hence necessitating a combination of degradation and secretion of putrescine released from the nucleoid. As was the case for pyruvate, the transient increase in extracellular lactate concentration, albeit to a much lower level than that observed for pyruvate (0.2 mmol lactate h^−1^ compared with 4 mmol pyruvate h^−1^), was not accompanied by an increase in the intracellular lactate concentration, approximately 8 mM before and after transition (figures 1 and 2). This suggests that, although there is an increased flux from pyruvate to lactate through the action of lactate dehydrogenase(s) after aeration, this does not provide a significant outlet for pyruvate accumulating as a result of the inactivation of PFL during the acute phase of adaptation to aerobic conditions.

**Figure 3.**
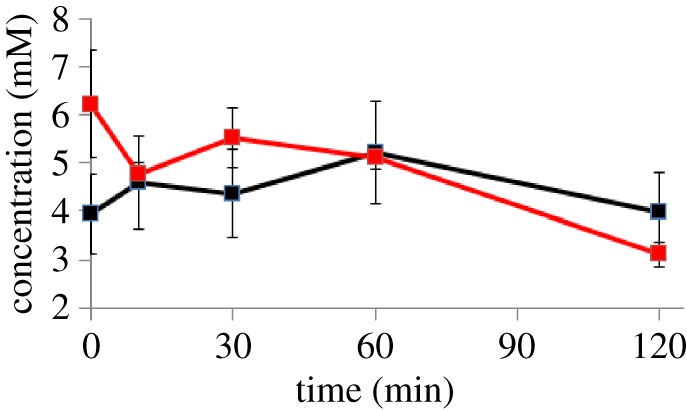
Comparison of intracellular pyruvate concentrations following introduction of oxygen to anaerobic culture, as measured by NMR (black) and using a pyruvate assay (red). Error bars for the assay are the standard deviation from three independent measurements, and for the NMR assay are 20.6% (electronic supplementary material, table S1).

The most surprising intracellular metabolite is pyruvate: we had expected a large transient rise in pyruvate concentration. The analysis in the previous section shows that pyruvate metabolism almost stops following introduction of oxygen, and that extracellular pyruvate rises rapidly. Normally, the *E. coli* cell maintains a high ratio of intracellular to extracellular pyruvate [29]. We had therefore expected that the pyruvate export would be driven by a high intracellular concentration, of more than 100 mM, but in fact it remained remarkably constant at about 6 mM. The concentration was checked using a standard pyruvate assay based on pyruvate oxidase, which gave similar results (figure 3). Calculations of the activity of the PdhR transcription factor (which is regulated by pyruvate concentrations) also implied an increase in pyruvate concentration [6,30].

The observation that pyruvate appears in the culture medium at a rate of approximately 4 mmol pyruvate h^−1^ in the absence of intracellular pyruvate accumulation was grossly different from expectations (see above). This raised the question of how and why the cell secretes such a large amount of pyruvate without increasing the intracellular concentration. Glucose crosses the inner membrane of *E. coli* via a phosphotransferase system that couples phosphotransfer from phosphoenolpyruvate (PEP) to glucose resulting in glucose-6-phosphate and pyruvate being generated in proximity to the cell membrane [31]. However, this process operates before and after the introduction of air, and the C balances are consistent with this membrane-proximal pyruvate entering fermentative metabolism before perturbation. Therefore, the fate of pyruvate during the acute phase of the transition to aerobic respiration must differ from the anaerobic steady state implying that there is some adaptation at the membrane to permit pyruvate excretion. A possible explanation for the high concentration of extracellular pyruvate in the absence of an increase in intracellular accumulation is that pyruvate is secreted from the cell as soon as it appears in the cytoplasm. If pyruvate is secreted without appearing in the cytoplasm then the enzyme(s) that produce it might relocate from the cytoplasm to the cell membrane. The location of the major pyruvate kinase (PykF) was, therefore, investigated.

### 2.4. Fluorescent labelling of pyruvate kinase F

Pyruvate kinase is the enzyme that converts PEP plus ADP to pyruvate plus adenosine triphosphate (ATP). It is the final reaction in the glycolytic pathway and a major source of ATP for the cell in anaerobic growth [32]. *Escherichia coli* has two pyruvate kinase enzymes: pyruvate kinases A and F (PykA or pyruvate kinase II and PykF or pyruvate kinase I, respectively), coded by the genes *pykA* and *pykF* [27]. Under many environmental conditions, both pyruvate kinase enzymes are expressed [33]. PykF is involved in controlling glycolytic flux via allosteric activation by fructose 1,6-bisphosphate and inhibition by ATP and succinyl-CoA; these allosteric effectors do not alter the homotetrameric state of the protein [26]. However, X-ray crystal structures indicate that the 12 domains that make up the PykF tetramer change orientation such that the domain interfaces couple changes in tertiary and quaternary structure in response to occupation of the fructose 1,6-bisphosphate and substrate binding sites [34,35]. The ability of PykF to undergo major structural changes in response to metabolic signals suggests that such changes might contribute to localizing PykF in the cytoplasmic or membrane fractions of *E. coli*. We therefore investigated PykF in this study.

It was considered important to keep the regulation of *pykF* the same as found in wild-type cells. Therefore, the chromosomal copy of *pykF* was removed and replaced by a tagged copy, using a methodology based on *λ* Red [36]. A two-step polymerase chain reaction (PCR) method was used to produce the desired DNA sequence, described in more detail in the electronic supplementary material. Two different tagged versions of *pykF* were generated: a version encoding a C-terminal GFP, described in this section, and a version encoding a C-terminal 3 × FLAG tag, described in the next section.

Bacteria expressing the chromosomal PykF-GFP fusion were grown to anaerobic steady state and perturbed by aeration. The cultures were sampled at various times, quenched using cold ethanol/saline, and simultaneously fixed using paraformaldehyde–glutaraldehyde. Images were taken of cells at anaerobic steady state, and 5 and 10 min after perturbation (electronic supplementary material, figure S3).

The apparent cell widths at 0, 5 and 10 min after introduction of oxygen were 11.4 ± 0.7, 11.3 ± 0.6 and 11.5 ± 1.4 pixels, respectively (*n *= 30 in each case), indicating that cell shape did not change upon aeration. Furthermore, the total fluorescence intensity did not change, showing that the total amount of PykF remained constant over this time period. No change in the distribution of fluorescence across the cell could be observed (electronic supplementary material, figure S4 and supplementary text). Analysis of the way in which fluorescence intensity is diffused by a real cell showed that this result is not unexpected, and hence that GFP fluorescence is unable to provide the spatial resolution required to detect any changes in location of PykF.

### 2.5. FLAG tag labelling of pyruvate kinase F

**Figure 4.**
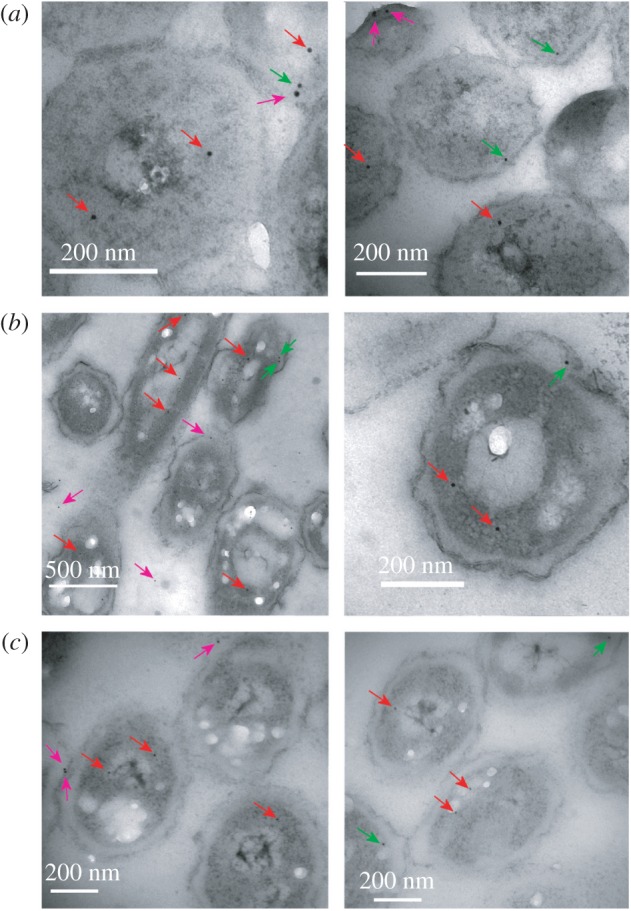
Typical transmission electron microscopy images of *E. coli* cells treated with nanogold-conjugated FLAG antibody to reveal locations of PykF-FLAG. (*a*) 0 min, (*b*) 2 min and (*c*) 30 min. Arrows mark nanogold particles judged as cytoplasmic (red), inner membrane (green) and outside the inner membrane (purple).

As fluorescence has insufficient spatial resolution, we tried a different approach, namely FLAG tagging. Although there are many reports of FLAG-tagged bacterial proteins, most of these use the tagging for pull-downs or western blotting. We have only identified a small number of publications in which FLAG tags have been used to localize proteins within bacteria, either using immunofluorescence [37–39] or nanogold particles [40]. As in our case the change in localization is likely to be rather small and transient, and as fluorescent labelling had previously been unable to distinguish between the two, we opted for nanogold labelling. The chromosomal copy of *pykF* was replaced by a C-terminally 3 × FLAG-tagged version. Cells were then treated in an identical way as described in the previous section. They were grown anaerobically to steady state, after which oxygen was introduced and cells were sampled at different time points (0, 2, 5 and 30 min), rapidly quenched and simultaneously fixed, and embedded in resin. They were then sectioned, stained and probed with nanogold-conjugated anti-FLAG antibodies. The location of PykF-FLAG is indicated by the nanogold particles (figure 4).

**Figure 5.**
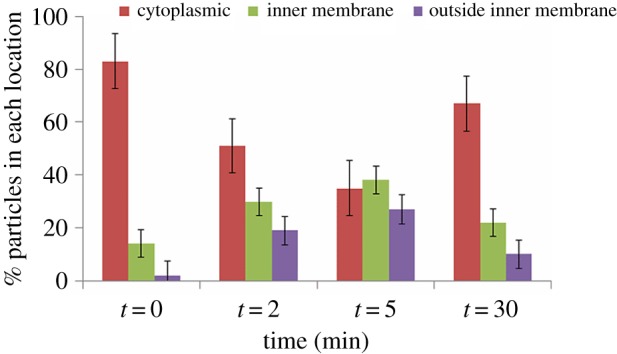
Location of nanogold particles at different times after the introduction of oxygen. The number of particles counted is 138, 216, 164 and 178 at 0, 2, 5 and 30 min, respectively; error bars show the standard error from repeated blind-independent counting of the same images.

There is no gross change in cell morphology from 0 to 30 min, confirming that pyruvate is not sequestered into vesicles. We also note that transcriptomics shows no significant change in *pykF* transcripts, and that the fluorescence experiments show no change in PykF-GFP concentration over this time scale, showing that total amounts of PykF remain constant. The locations of nanogold particles were counted at each time point. In total, 696 particles were observed, and their positions were classified into cytoplasmic, adjacent to the cell membrane, or outside the cell membrane (i.e. periplasmic or outside the cell). The results indicate a dramatic redistribution of particles from cytoplasm to membrane (figure 5), starting within 2 min of aeration and almost recovering to baseline by 30 min. We therefore conclude that at least 50% of PykF relocates to the cell membrane within 5–10 min of aeration, but then returns to the cytoplasm over the next 30–60 min.

It was a surprise to see particles outside the cell membrane. Some particles external to the cell are to be expected due to random labelling, and labelling of PykF-FLAG that has leaked from damaged cells. However, there were significant numbers of particles in apparently periplasmic locations (figure 4). It is possible that these were in fact on the cell membrane, but that the cell membrane is extended towards the cell wall at these places. If so, most of the ‘outside inner membrane’ particles (figure 5) should be classified as inner membrane, further increasing the effect of aerobiosis on PykF distribution. The quality of the results produced by FLAG tags imaged by nanogold-conjugated antibodies holds the promise that this methodology may be more widely applicable in bacteria, particularly where super-resolution microscopy is inappropriate.

## 3. Discussion

We have developed and tested a method for rapid quenching of metabolism in *E. coli* and extracting metabolites for measurement by NMR. The method leads to minimal leakage of cell contents, and cools cells to 1°C within 2 s of sampling and to −5°C within 1 min, thereby quenching metabolism effectively and leading to reliable and reproducible quantitation of metabolites. The error in measurement of intracellular metabolite concentrations is 20% from all sources combined, which although large is acceptable, and probably no larger than the error in measurement of absolute concentrations due to the assumptions made in this process. The method is simple, cheap and effective, and should be applicable to a wide range of unicellular bacteria.

Measurement of extracellular metabolites confirms previous results and is consistent with immediate cessation of anaerobic metabolism on introduction of oxygen. However, the transient increase of extracellular pyruvate and lactate, and the continued secretion of acetate, show that aerobic metabolism does not catch up immediately, taking up to 2 h to reach full activity. These results are in complete agreement with earlier transcriptomic measurements [5].

The most striking result presented here is that intracellular metabolites show very little change in concentration during the transition from anaerobic to aerobic growth. Most metabolite concentrations move steadily from anaerobic to aerobic values. This is despite the fact that PFL, which is responsible for anaerobic metabolism of pyruvate and is thus a pivotal enzyme for anaerobic carbon flux when cells are growing on glucose, is inactivated rapidly on aeration, while its aerobic replacement PDHC is not fully active until at least 60 min into the transition. We might, therefore, expect a large transient increase in intracellular pyruvate, but this was not the case, with intracellular pyruvate concentrations remaining remarkably stable (figure 2).

The results presented here suggest that PykF, which is the last enzyme in the glycolytic pathway and the one responsible for converting PEP plus ADP into pyruvate plus ATP, is translocated from the cytoplasm to the cell membrane on introduction of oxygen. The correlation between PykF relocation and pyruvate secretion is striking, although it does not constitute proof of a causal link. Movement of PykF from cytoplasm to membrane does not require a transport mechanism and could occur by simple Brownian diffusion directed by a change in affinity for the pyruvate transporter (caused for example by an allosteric structural change). For example, the cell division inhibitor MinC relocates completely from one pole of the cell to the other in about 25 s, stimulated only by binding to targets at the cell poles [41].

It has been reported that pyruvate excretion by bacteria is responsive to environmental conditions (e.g. acid pH, O_2_-limitation, growth rate inhibition and metabolic bottlenecks) and contributes to biological fitness and virulence [42–46]. Thus, inhibition of growth of *Aerobacter aerogenes* by antibiotics resulted in pyruvate accumulation, perhaps as a consequence of a lower demand for C_4_-units in biosynthesis [46]. Furthermore, several strains of marine bacteria were found to excrete pyruvate when grown on glucose under aerobic conditions, suggesting that glycolytic flux exceeded the capacity of the citric acid cycle to process pyruvate [44]. More recently, pyruvate secretion by the pathogen *Yersinia pseudotuberculosis* has been suggested to contribute to virulence [47]. Thus, it is tempting to speculate that the localization of PykF to facilitate excretion of pyruvate might be stimulated not only in response to the anaerobic–microaerobic transition reported here but by many other conditions in a wide range of bacterial species.

Here, we have looked at the location only of PykF and have not considered other enzymes in the glycolytic pathway. The high rate of glycolysis in these cells continuing throughout the transition means that any kinetic barrier within the glycolytic pathway would lead to a rapid build-up of glycolytic intermediates, which was not seen, implying that there may be a relocation of a complex of glycolytic enzymes (a metabolon) not just PykF. Interestingly, an electrophoretic analysis of *E. coli* protein complexes suggested that PykF could exist as a homomultimeric complex in the cytoplasm but also associated with the cytoplasmic membrane [48]. The same study indicated that phosphoglycerate kinase, glyceraldehyde-3-phosphate dehydrogenase and lipoamide dehydrogenase could also be found in the membrane fraction of *E. coli*, suggesting that a larger glycolytic complex could relocate from the cytoplasm to the membrane in response to growth conditions. The search for glycolytic metabolons has a long history going back at least to 1977 [49], being popularized by Srere from the 1980s onwards [50–52]. The concept of the metabolon has stirred up more than a little heat, with much expectation but little experimental verification [53]. More recently, experimental evidence for metabolons has appeared [54], including evidence for a glycolytic metabolon in plants [55], and it is becoming clear that metabolons do exist but are dynamic rather than static, being induced when there is a metabolic need, and they almost always assemble on membranes (or on other surfaces such as the cytoskeleton) [56]. In erythrocytes, glycolytic enzymes associate with the anion transporter band 3, in areas where ATP is consumed, forming an ordered complex [57,58]. We are currently investigating whether other enzymes relocate together with PykF, and thus whether there might be a glycolytic metabolon in *E. coli*, which assembles on the cell membrane associated with a pyruvate transporter and is induced by the metabolic stress resulting from aeration.

We note that the relocation of PykF, such that pyruvate is exported from the cell rather than building up intracellularly, implies that *in silico* models of *E. coli* metabolism [32,59] could be incomplete and may need modification. If *E. coli* can smoothly regulate a homeostatic system like this, one wonders how many other such systems there are still to be uncovered.

The fact that concentrations of metabolites in *E. coli* remain so stable during the anaerobic/aerobic transition, when such major changes in cellular flux are occurring, is clearly not a coincidence. The cells must have evolved a detailed regulatory mechanism for ensuring homeostasis, most of which is so far not understood. There has clearly been evolutionary pressure to maintain homeostasis within very tight limits, implying that it has strong functional benefits for the cell.

## 4. Material and methods

### 4.1. Cell growth

Cultures of *E. coli* K-12 strain MG 1655 were grown in a 1 l capacity Labfors 3 chemostat (Infors) at 37°C with stirring at 400 r.p.m. The pH was maintained at 7.0 by automatic titration with 1 M KOH. A carbon-limited defined medium was used, which contains 20 mM glucose as the carbon source. It was based on that of Evans *et al.* [60] except that 2 mM nitrilotriacetic acid was used as the metal chelator instead of citrate. Growth medium was fed at a dilution rate of 0.2 h^−1^ in order to create a steady state. Anaerobic cultures were sparged with N_2_/CO_2_ 95/5 at 0.4 l min^−1^, and the switch to aerobiosis used air at 0.4 l min^−1^. Dissolved oxygen levels were measured with a TruDO dissolved oxygen sensor (Finesse).

### 4.2. Quenching and analysis

The method is a modification of that of Spura *et al.* [15]. A quenching solution containing 20 ml of 40% ethanol (v/v) and 0.8% NaCl (w/v) in a 50 ml tube was cooled to −35°C in a dry ice/isopropanol bath. This is the lowest temperature that can be achieved with this mixture without it solidifying. A 20 ml sample was removed from the chemostat by syringe, and mixed rapidly with the quenching solution. The solution was left in the isopropanol bath for a further minute until the temperature reached −5°C, as measured by a thermometer. It was essential to stir the cooling solution continuously with the thermometer to avoid local freezing and cell damage. Once at −5°C, the tube was transferred to an ice/salt mixture and centrifuged in a precooled centrifuge (Beckman Avanti) at −11°C (5 min, 3940*g*). The supernatant was frozen until analysis, while the pellet was cooled immediately in liquid nitrogen, and metabolites were extracted by three freeze–thaw cycles [61]. Methanol at −80°C was added, the solution was vortexed, and cells were frozen in liquid nitrogen, thawed and centrifuged [62]. Supernatant was pooled and lyophilized. For NMR analysis, 500 µl of D_2_O plus 5 µl of a 10 mM stock of *d*_4_-trimethylsilyl propionate (TSP; standard) was added.

NMR spectra were acquired with a Bruker DRX-500 at 298 K. The residual water signal was reduced by presaturation for 2 s. The acquisition time was 2.5 s, giving a total recycle time of 4.5 s. A 1 Hz line broadening and polynomial baseline correction was applied. Metabolites were identified by spiking with standards, which were also used to quantitate concentrations. Concentrations were measured by integration (using the Bruker Topspin program) and compared with TSP.

Intracellular pyruvate concentration was measured using an Enzychrome™ pyruvate assay kit (Bioassay Systems).

### 4.3. DNA methods

The chromosomal copy of *pykF* was replaced by *FLAG*-tagged or *gfp*-tagged versions by a two-step PCR procedure based on *λ* Red methodology. Details are given in the electronic supplementary material.

### 4.4. Fluorescence microscopy

Cells were grown in the chemostat as above, sampled into a quench/fix solution containing 40% ethanol (v/v), 15% paraformaldehyde (v/v), 25% glutaraldehyde (v/v) and 0.8% saline (w/v) at −35°C, and further cooled to −5°C. Samples were kept at 4°C in a shaker for 1 h to complete fixation, and centrifuged and washed in phosphate-buffered saline three times. Cells were resuspended in GTE solution (50 mM glucose, 10 mM EDTA, 20 mM Tris pH 7.5), and 5 µl samples were attached to a poly-l-lysine coated microscope slide, dried and washed. Images were acquired on an Olympus BX61 upright microscope with a 100× (NA 1.4) oil objective lens. Images were acquired as *z*-stacks, and the central plane was used for image analysis. Python scripts were used within Fiji [63] to select cross sections across cells and measure pixel intensities, interpolating values from neighbouring pixels when the cross section traversed a pixel boundary. Cross-sectional intensities were analysed using Excel (Microsoft). Background intensities were subtracted, and apparent cell edges determined by fitting straight lines to the rise and fall of fluorescence.

### 4.5. Transmission electron microscopy

Cells were grown, sampled, quenched and fixed as described above. They were washed successively with buffer, aqueous ethanol, ethanol and epoxypropane, embedded in resin (araldite CY 212/dodecenyl succinic anhydride/benzyldimethylamine), sectioned at approximately 85 nm (Lowycril Mono-step HM 20 Resin Agar Scientific, Stansted, UK) and deposited onto Fomvar-coated nickel grids. They were labelled for 2 h with primary rabbit anti-FLAG antibody (Sigma, diluted 50× with TBS (50 mM Tris, 150 mM NaCl) pH 7.4 containing 0.1% bovine serum albumin (w/v)), washed twice and labelled with secondary goat anti-rabbit antibodies conjugated with 10 nm gold particles (Biocell), diluted 50× in TBS pH 8.2 containing 0.1% bovine serum albumin (w/v). They were then washed, negatively stained with 5% uranyl acetate (w/v) for 2 min, washed and stained in 0.5% alkaline lead citrate (w/v), pH 12, for 2 min. Grids were imaged using a FEI Tecnai transmission electron microscope at 80 kV, and recorded using a Gatan multiscan 600 digital camera. All images were processed using a range of image adjustments to confidently locate nanogold particles. To avoid user bias, images were counted blind, i.e. without reference to the time at which the image was taken. Images were counted several times independently in order to determine the reliability of the categorizations into cytoplasmic, inner membrane and other.

## Supplementary Material

supplementary_material contains four additional figures, a discussion of resolution in the fluorescence microscopy, a table, some experimental details, and a few references

## Supplementary Material

files to zip.zip

## Data accessibility

Supporting data have been stored in the Dryad archive at http://dx.doi.org/10.5061/dryad.1n542.
