# Supplementary material for: Homeostasis of metabolites in Escherichia coli on transition from anaerobic to aerobic conditions and the transient secretion of pyruvate
Source: R Soc Open Sci. 2016 Aug 24;3(8):160187. doi: 10.1098/rsos.160187 (PMC5108944; doi:10.1098/rsos.160187)
Supplement: files to zip.zip [file rsos160187supp2.zip › files to zip.tar!/figure legends.doc]

**Figure legends**

**Figure 1.** Concentrations of extracellular metabolites following introduction of oxygen to anaerobic culture. Errors are standard deviations (*n*=3 independent cultures). For formate, succinate and ethanol, the dashed lines show a best fit to a decay of 0.2 h^-1^; for acetate, the dashed line is constant.

**Figure 2.** Concentrations of intracellular metabolites following introduction of oxygen to anaerobic culture. Errors are standard deviations (*n*=3 independent cultures). For formate and succinate, the dashed lines show a best fit to a decay of 0.2 h^-1^; for acetate, the dashed line is constant.

**Figure 3.** Comparison of intracellular pyruvate concentrations following introduction of oxygen to anaerobic culture, as measured by NMR (black) and using a pyruvate assay (red). Error bars for the assay are the standard deviation from 3 independent measurements, and for the NMR assay are 20.6% (Table S1).

**Figure 4.** Typical transmission electron microscopy images of *E. coli* cells treated with nanogold-conjugated FLAG antibody to reveal locations of PykF-FLAG. (a) 0 min, (b) 2 min, (c) 30 min. Arrows mark nanogold particles judged as cytoplasmic (red), inner membrane (green) and outside the inner membrane (purple).

**Figure 5.** Location of nanogold particles at different times after the introduction of oxygen. The number of particles counted is 138, 216, 164, 178 at 0, 2, 5 and 30 min respectively; error bars show the standard error from repeated blind independent counting of the same images.
