# Supplementary material for: Homeostasis of metabolites in Escherichia coli on transition from anaerobic to aerobic conditions and the transient secretion of pyruvate
Source: R Soc Open Sci. 2016 Aug 24;3(8):160187. doi: 10.1098/rsos.160187 (PMC5108944; doi:10.1098/rsos.160187)
Supplement: files to zip.zip [file rsos160187supp2.zip › files to zip.tar!/references.doc]

**References**

1.	Guest JR, Green J, Irvine AS, Spiro S. 1996. In *Regulation of gene expression in Escherichia coli.* (eds Lin ECC, Lynch AS), pp. 317-342. Austin, TX: R. G. Landes & Co.

2.	Murarka A, Clomburg JM, Moran S, Shanks JV, Gonzalez R. 2010 Metabolic analysis of wild-type *Escherichia coli* and a pyruvate dehydrogenase complex (PDHC)-deficient derivative reveals the role of PDHC in the fermentative metabolism of glucose. *J. Biol. Chem.* **285**, 31548-31558.

3.	Hoskisson PA, Hobbs G. 2005 Continuous culture - making a comeback? *Microbiol.* **151**, 3153-3159.

4.	Schaub J, Reuss M. 2008 *In vivo* dynamics of glycolysis in *Escherichia coli* shows need for growth-rate dependent metabolome analysis. *Biotechnol. Progress* **24**, 1402-1407.

5.	Partridge JD, Scott C, Tang Y, Poole RK, Green J. 2006 *Escherichia coli* transcriptome dynamics during the transition from anaerobic to aerobic conditions. *J. Biol. Chem.* **281**, 27806-27815.

6.	Trotter EW, Rolfe MD, Hounslow AM, Craven CJ, Williamson MP, Sanguinetti G, Poole RK, Green J. 2011 Reprogramming of *Escherichia coli* K-12 metabolism during the initial phase of transition from an anaerobic to a micro-aerobic environment. *Plos One* **6**, e25501.

7.	Partridge JD, Sanguinetti G, Dibden DP, Roberts RE, Poole RK, Green J. 2007 Transition of *Escherichia coli* from aerobic to micro-aerobic conditions involves fast and slow reacting regulatory components. *J. Biol. Chem.* **282**, 11230-11237.

8.	Takahashi N, Abbe K, Takahashi-Abbe S, Yamada T. 1987 Oxygen sensitivity of sugar metabolism and interconversion of pyruvate formate-lyase in intact cells of *Streptococcus mutans* and *Streptococcu sanguis*. *Infect. Immun.* **55**, 652-656.

9.	Wagner AF, Schultz S, Bomke J, Pils T, Knappe J. 2001 YfiD of *Escherichia coli* YO61 of bacteriophage T4 as autonomous glycyl radical cofactors reconstituting the catalytic center of oxygen-fragmented pyruvate formate lyase. *Biochim. Biophys. Res. Commun.* **285**, 456-462.

10.	de Koning W, van Dam K. 1992 A method for the determination of changes of glycolytic metabolites in yeast on a subsecond time scale using extraction at neutral pH. *Anal. Biochem.* **204**, 118-123.

11.	Buchholz A, Hurlebaus J, Wandrey C, Takors R. 2002 Metabolomics: quantification of intracellular metabolite dynamics. *Biomol. Eng.* **19**, 5-15.

12.	Ritter JB, Genzel Y, Reichl U. 2008 Simultaneous extraction of several metabolites of energy metabolism and related substances in mammalian cells: Optimization using experimental design. *Anal. Biochem.* **373**, 349-369.

13.	Link H, Anselment B, Weuster-Botz D. 2008 Leakage of adenylates during cold methanol/glycerol quenching of *Escherichia coli*. *Metabolomics* **4**, 240-247.

14.	Bolten CJ, Kiefer P, Letisse F, Portais J-C, Wittmann C. 2007 Sampling for metabolome analysis of microorganisms. *Anal. Chem.* **79**, 3843-3849.

15.	Spura J, Reimer LC, Wieloch P, Schreiber K, Buchinger S, Schomburg D. 2009 A method for enzyme quenching in microbial metabolome analysis successfully applied to gram-positive and gram-negative bacteria and yeast. *Anal. Biochem.* **394**, 192-201.

16.	Gonzalez B, François J, Renaud M. 1997 A rapid and reliable method for metabolite extraction in yeast using boiling buffered ethanol. *Yeast* **13**, 1347-1355.

17.	Kayser A, Weber J, Hecht V, Rinas U. 2005 Metabolic flux analysis of *Escherichia coli* in glucose-limited continuous culture. I. Growth-rate dependent metabolic efficiency at steady state. *Microbiology* **151**, 693-706.

18.	Maharjan RP, Ferenci T. 2003 Global metabolite analysis: the influence of extraction methodology on metabolome profiles of *Escherichia coli*. *Anal. Biochem.* **313**, 145-154.

19.	Bennett BD, Kimball EH, Gao M, Osterhout R, Van Dien SJ, Rabinowitz JD. 2009 Absolute metabolite concentrations and implied enzyme active site occupancy in *Escherichia coli*. *Nature Chem. Biol.* **5**, 593-599.

20.	Taymaz-Nikerel H, Borujeni AE, Verheijen PJT, Hiejnen JJ, van Gulik WM. 2010 Genome-derived minimal metabolic models for *Escherichia coli* MG1655 with estimated *in vivo* respiratory ATP stoichiometry. *Biotechnol. Bioeng.* **107**, 369-381.

21.	Churchward G, Estiva E, Bremer H. 1981 Growth rate dependent control of chromosome replication initiation in *Escherichia coli*. *J. Bact.* **145**, 1232-1238.

22.	Loferer-Krossbacher M, Klima J, Psenner R. 1998 Determination of bacterial cell dry mass by transmission electron microscopy and densitometric image analysis. *Applied Environ. Microbiol.* **64**, 688-694.

23.	Neidhart FC, Umbarger HE. 1996 Chemical composition of *Escherichia coli*. In *Escherichia coli and Salmonella: cellular and molecular microbiology.* (eds Neidhart FC, Curtis III R, Ingraham JL, Lin ECC, Low KB, Magasanik B, Reznikoff WS, Riley M, Schaechhter M, Umbarger HE), pp. 13-28 2nd edn. Washington, D. C.: American Society for Microbiology.

24.	Kubitschek HE. 1969 Growth during bacterial cell cycle: Analysis of cell size distribution. *Biophys. J.* **9**, 792-809.

25.	Dittrich CR, Bennett GN, San KY. 2005 Characterization of the acetate-producing pathways in *Escherichia coli*. *Biotechn. Progr.* **21**, 1062-1067.

26.	Kim Y, Ingram LO, Shanmugam KT. 2008 Dihydrolipoamide dehydrogenase mutation alters the NADH sensitivity of pyruvate dehydrogenase complex of *Escherichia coli* K-12. *J. Bacteriol.* **190**, 3851-3858.

27.	Snoep JL, de Graef MR, Westphal AH, de Kok A, de Mattos MJT, Neijssel OM. 1993 Differences in sensitivity to NADH of purified pyruvate dehydrogenase complexes of *Enterococcus faecalis, Lactococcus lactis, Azotobacter vinelandii* and *Escherichia coli*: Implications for their activities in vivo. *FEMS Microbiol. Letts* **114**, 279-283.

28.	Alexeeva S, de Kort B, Sawers G, Hellingwerf KJ, de Mattos MJT. 2000 Effects of limited aeration and of the ArcAB system on intermediary pyruvate catabolism in *Escherichia coli*. *J. Bacteriol.* **182**, 4934-4940.

29.	Lang VJ, Leystra-Lantz C, Cook RA. 1987 Characterization of the specific pyruvate transport system in *Escherichia col*i K-12. *J. Bact.* **169**, 380-385.

30.	Rolfe MD, Ocone A, Stapleton MR, Hall S, Trotter EW, Poole RK, Sanguinetti G, Green J. 2012 Systems analysis of transcription factor activities in environments with stable and dynamic oxygen concentrations. *Open Biol.* **2**, 120091.

31.	Postma PW, Lengeler JW, Jacobson GR. 1993 Phosphoenolpyruvate:carbohydrate phosphotransferase systems of bacteria. *Microbiol. Rev.* **57**, 543-594.

32.	McCloskey D, Gangoiti JA, King ZA, Naviaux RK, Barshop BA, Palsson BO, Feist AM. 2014 A model-driven quantitative metabolomics analysis of aerobic and anaerobic metabolism in *E. coli* K-12 MG1655 that is biochemically and thermodynamically consistent. *Biotechnol. Bioeng.* **111**, 803-815.

33.	Ponce E, Flores N, Martinez A, Valle F, Bolívar F. 1995 Cloning of the two pyruvate kinase isoenzyme structural genes from *Escherichia coli*: The relative roles of these enzymes in pyruvate biosynthesis. *J. Bact.* **177**, 5719-5722.

34.	Mattevi A, Valentini G, Rizzi M, Speranza ML, Bolognesi M, Coda A. 1995 Crystal structure of *Escherichia coli* pyruvate kinase type I: Molecular basis of the allosteric transition. *Structure* **3**, 729-741.

35.	Valentin G, Chiarelli L, Fortin R, Speranza ML, Galizzi A, Mattevi A. 2000 The allosteric regulation of pyruvate kinase: A site-directed mutagenesis study. *J. Biol. Chem.* **275**, 18145-18152.

36.	Datsenko KA, Wanner BL. 2000 One-step inactivation of chromosomal genes in *Escherichia coli* K-12 using PCR products. *Proc. Natl. Acad. Sci. USA* **97**, 6640-6645.

37.	Orillard E, Radicella JP, Marsin S. 2011 Biochemical and cellular characterization of *Helicobacter pylori* RecA, a protein with high-level constitutive expression. *J. Bacteriol.* **193**, 6490-6497.

38.	Fukushima T, Afkham A, Kurosawa S-I, Tanabe T, Yamamoto H, Sekiguchi J. 2006 A new D,L-Endopeptidase gene product, YojL (renamed CwlS), plays a role in cell separation with LytE and LytF in *Bacillus subtilis*. *J. Bacteriol.* **188**, 5541-5550.

39.	Taghbalout A, Yang Q, Arluison V. 2014 The *Escherichia coli* RNA processing and degradation machinery is compartmentalized within an organized cellular network. *Biochem. J.* **458**, 11-22.

40.	Beznosov SN, Pyatibratov MG, Veluri PS, Mitra S, Fedorov OV. 2013 A way to identify archaellins in *Halobacterium salinarum* archaella by FLAG-tagging. *Central Europ. J. Biol.* **8**, 828-834.

41.	Meinhardt H, de Boer PAJ. 2001 Pattern formation in *Escherichia coli*: A model for the pole-to-pole oscillations of Min proteins and the localization of the division site. *Proc. Natl. Acad. Sci. USA* **98**, 14202-14207.

42.	Nakata HM. 1963 Effect of pH on intermediates produced during growth and sporulation of *Bacillus cereus*. *J. Bacteriol.* **86**, 577-581.

43.	O'Brien RW. 1975 Induction of citrate lyase in *Enterobacter cloacae* grown under aerated conditions and its effects on citrate metabolism. *J. Bacteriol.* **124**, 1084-1088.

44.	Ruby EG, Nealson KH. 1977 Pyruvate production and excretion by luminous marine bacteria. *Applied Environ. Microbiol.* **34**, 164-169.

45.	Steinbüchel A, Schlegel HG. 1989 Excretion of pyruvate by mutants of *Alcaligenes eutrophus*, which are impaired in the accumulation of poly(β-hydroxybutyric acid) (PHB), under conditions permitting synthesis of PHB. *Applied Microbiol. Biotechnol.* **31**, 168-175.

46.	Webb M. 1968 Pyruvate accumulation in growth-inhibited cultures of *Aerobacter aerogenes*. *Biochem. J.* **106**, 375-380.

47.	Bücker R, Heroven AK, Becker J, Dersch P, Wittmann C. 2014 The pyruvate-tricarboxylic acid cycle node: A focal point of virulence control in the enteric pathogen *Yersinia pseudotuberculosis*. *J. Biol. Chem.* **289**, 30114-30132.

48.	Lasserre J-P, Beyne E, Pyndiah S, Lapaillerie D, Claverol S, Bonneu M. 2006 A complexomic study of *Escherichia coli* using two-dimensional blue native/SDS polyacrylamide gel electrophoresis. *Electrophoresis* **27**, 3306-3321.

49.	Welch GR. 1977 Role of organized multienzyme systems in cellular metabolism. General synthesis. *Progr. Biophys. Mol. Biol.* **32**, 103-191.

50.	Srere PA. 1987 Complexes of sequential metabolic enzymes. *Ann. Rev. Biochem.* **56**, 89-124.

51.	Ovádi J, Srere PA. 1992 Channel your energies. *Trends in Biochemical Sciences* **17**, 445-447.

52.	Srere PA. 2000 Macromolecular interactions: tracing the roots. *Trends Biochem. Sci.* **25**, 150-153.

53.	Knowles JR. 1991 Calmer waters in the channel? *J. Theor. Biol.* **152**, 53-55.

54.	Zhao H, Chiaro CR, Zhang L, Smith PB, Chan CY, Pedley AM, Pugh RJ, French JB, Patterson AD, Benkovic SJ. 2015 Quantitative analysis of purine nucleotides indicates that purinosomes increase *de novo* purine biosynthesis. *J. Biol. Chem.* **290**, 6705-6713.

55.	Graham JWA, Williams TCR, Morgan M, Fernie AR, Ratcliffe RG, Sweetlove LJ. 2007 Glycolytic enzymes associate dynamically with mitochondria in response to respiratory demand and support substrate channeling. *Plant Cell* **19**, 3723-3738.

56.	Williamson MP. 2011 *How Proteins Work*. New York: Garland Science.

57.	Puchulu-Campanella E, Chu H, Anstee DJ, Galan JA, Tao WA, Low PS. 2013 Identification of the components of a glycolytic enzyme metabolon on the human red blood cell membrane. *J. Biol. Chem.* **288**, 848-858.

58.	Campanella ME, Chu HY, Low PS. 2005 Assembly and regulation of a glycolytic enzyme complex on the human erythrocyte membrane. *Proc. Natl. Acad. Sci. USA* **102**, 2402-2407.

59.	Ederer M, Steinsiek S, Stagge S, Rolfe MD, Ter Beek A, Knies D, de Mattos MJT, Sauter T, Green J, Poole RK *et al*. 2014 A mathematical model of metabolism and regulation provides a systems-level view of how *Escherichia coli* responds to oxygen. *Frontiers Microbiol.* **5**, 124.

60.	Evans CGT, Herbert D, Tempest DW. 1970 *The continuous cultivation of microorganisms. Part 2: Construction of a chemostat*. New York: Academic Press.

61.	Theobald U, Mailinger W, Reuss M, Rizzi M. 1993 *In vivo* analysis of glucose-induced fast changes in yeast adenine-nucleotide pool applying a rapid sampling technique. *Anal. Biochem.* **214**, 31-37.

62.	Faijes M, Mars AE, Smid EJ. 2007 Comparison of quenching and extraction methodologies for metabolome analysis of *Lactobacillus plantarum*. *Microbial Cell Factories* **6**, 27.

63.	Schindelin J, Arganda-Carreras I, Frise E, Kaynig V, Longair M, Pietzsch T, Preibisch S, Rueden C, Saalfeld S, Schmid B *et al*. 2012 Fiji: an open-source platform for biological image analysis. *Nature Methods* **9**, 676-682.
