# Supplementary material for: Homeostasis of metabolites in Escherichia coli on transition from anaerobic to aerobic conditions and the transient secretion of pyruvate
Source: R Soc Open Sci. 2016 Aug 24;3(8):160187. doi: 10.1098/rsos.160187 (PMC5108944; doi:10.1098/rsos.160187)
Supplement: files to zip.zip [file rsos160187supp2.zip › files to zip.tar!/supplementary_material.docx]

Homeostasis of metabolites in *Escherichia coli* on transition from anaerobic to aerobic conditions and the transient secretion of pyruvate

Nur Adeela Yasid, Matthew D. Rolfe, Jeffrey Green and Mike P. Williamson

*Supplementary material*

**Figure S1.** ^1^H NMR spectra of supernatants from *E. coli* cell suspensions quenched and centrifuged as described. Four typical supernatant spectra are superimposed, and compared to a sample spiked with glutamate (blue, bottom). Any glutamate leaking out of cells as a result of cell damage during the quenching would give rise to signals from glutamate, which is the most abundant metabolite in *E. coli* cells and is present at intracellular concentrations of 0.1 M [1]. No glutamate peaks are visible: the maximum concentration of glutamate present in the supernatants is determined by spectral noise and is 2.6 ± 1.2 μM. Assuming an intracellular volume of 0.5 fl, and the measured 1.1 x 10^9^ cells/ml in the original suspension, this implies that no more than 5% of intracellular glutamate has leaked out.

**Figure S2.** ^1^H NMR spectra of supernatant from *E. coli* grown at steady state (a) aerobically and (b) anaerobically. NTA: nitrilotriacetic acid buffer (2 mM). TSP: d_4_-trimethylsilylpropionate internal standard.

 **Figure S3.** Fluorescence microscopy images of *E. coli* cells containing PykF tagged with green fluorescent protein. Cells were quenched (left) 0 and (right) 5 min after introduction of oxygen.

Distribution of fluorescence labelling of pyruvate kinase F

We investigated whether there is any change in the apparent intensity of fluorescence across the cell width, indicating a time-dependent relocation of PykF. When cells are imaged by fluorescence microscopy, the fluorescence from each point in the cell is spread out by a point spread function, which for an ideal lens is known as an Airy disk, and can be approximated to a Gaussian function $I_{q}=I_{0}exp\left( \frac{-q^{2}}{2\left( m\lambda\right)^{2}} \right)$ , where *q* is the radial distance, λ is the wavelength, and *m* depends on the quality of the optical system, but is a minimum of 0.4 [2]. In our system, the best match to experimentally observed fluorescence corresponded to *m* = 0.6. This means that the image is more diffuse than the original sample. This was modelled for *E. coli* cells (Fig. S4), indicating that a 100% change from a uniform distribution across the cell to location only at the cell membrane would cause a rather subtle change in the distribution of fluorescence, amounting to a 20% decrease in intensity at the centre of the cell. We measured the distribution of fluorescence across the cell width. The mean fluorescence intensity in the centre of the cell (Fig. S4, green bar) compared to the total intensity across the cell at 0, 5 and 10 min was 6.8 ± 1.0, 6.8 ± 0.8 and 6.8 ± 0.9% (*n*=30) respectively, showing no measurable change in the distribution. Modelling indicated that a rather more sensitive measure is the ratio of fluorescence intensity inside the ‘true’ cell width to the fluorescence intensity outside the true cell width (Fig. S4, blue/red bars). At 0, 5 and 10 min, these ratios were 1.75 ± 0.31, 1.70 ± 0.35 and 1.83 ± 0.37 (*n*=30), again showing no significant change. We therefore concluded that fluorescence is unable to provide the spatial resolution required to detect any changes in location of PykF.

**Figure S4**. Observed (centre panel, t=0, three representative cells) and expected (left and right panels) fluorescence distribution across the cell width, using a Gaussian point spread function. True cell boundary is indicated by the shaded area; the brown line shows expected distribution if the fluorescence is distributed evenly across the cell width, while the green line shows the distribution when fluorescence is only present at the cell membrane. Left and right panels show simulations for m=0.4 and 0.6, representing an ideal lens and a real optical system respectively. Bars on the central panel indicate the measures used to study the distribution of fluorescence across the cell. The green bar shows the width of the ‘central’ part of the cell (for comparison of fluorescence intensity in the central part compared to total intensity). The blue and red bars denote the regions used to measure fluorescence inside and outside the true cell width, respectively.

**Table S1**

**Magnitude of sources of errors for NMR detection of metabolites from chemostat growths**

| Source of error | % error (total)^a^ | % error (individual) ^a^ |
| --- | --- | --- |
| Integration | 0.6 | 0.6 |
| Sampling | 1.8 | 1.2 |
| Biological variation (supernatant) | 2.1 | 0.3 |
| Quenching | 16.0 | 14.2 |
| Biological variation (intracellular) | 20.6 | 4.5 |

Errors were determined by repeated measurements, using different types of repeats to measure different sources of error.

*Integration error* is the error from processing and integrating the NMR raw data, and was estimated by repeated processing of identical FIDs. If the baseline is flat and signals are reasonably sharp, the biggest integration error comes from poor signal-to-noise [3], and was minimized by acquiring up to 1024 scans for the less sensitive intracellular measurements. Various automated methods were tried for setting integration limits, baseline correction and phasing, but the most reproducible results were obtained by doing these manually. The error reported is the standard deviation for measuring the relative intensity of two signals, expressed as a percentage of the peak intensity.

*Sampling error* is the error arising from independent sampling and handling of the same biological sample, and was determined by collecting several samples from the same culture at the same time and measuring them independently. This includes pipetting errors.

*Biological variation error*, as measured for supernatant (extracellular) samples, is the variation arising from the fact that different biological samples may be different. It was determined by preparing different steady-state cultures, starting from the same starter culture (stored at -80˚ in aliquots). The biological variation error for intracellular samples is likely to be much larger (and indeed is much larger), because one would expect that intracellular concentrations from different cultures vary more than extracellular ones, because of both natural variation and handling. Contamination of intracellular samples by the medium is a much bigger problem than contamination of extracellular samples by cell leakage, because intracellular volume is very small compared to the extracellular volume.

*Quenching error* comes from experimental variability in the quenching process. Because the supernatant volume is so much larger than the intracellular volume (and there is no observable leakage of metabolites), quenching error only affects intracellular measurements.

^a^The *total error* (column 2) was assumed to be the sum of the *individual errors* from the relevant different sources (column 3). Repeated processing and integration of the same dataset generates a total error which arises entirely from individual integration error. Therefore the total error for integration is the same as the individual error. The total error for repeated sampling of the same culture, followed by processing and integration of the results, is the sum of individual integration and sampling errors. The individual error for sampling can therefore be obtained by subtracting the individual integration error from the total sampling error, the difference being assumed to be only due to sampling error. The total error in measuring extracellular supernatants from different cultures is the sum of integration, sampling and (supernatant) biological variation errors. The individual error for extracellular biological variation was therefore obtained by subtracting the total sampling error (or equivalently, the sum of the individual sampling and integration errors). The total error in measuring intracellular metabolites in repeated samples of the same steady-state culture should be the sum of integration, sampling and quenching errors, while the error in measuring intracellular metabolites from different but repeated cultures is the sum of integration, sampling, quenching and biological variation error. The last two individual error values listed in the table are therefore a compromise, since the experimental data do not provide a completely consistent set of error values (implying that some of the errors may not be independent of each other).

Generation of *FLAG*- and *gfp*-tagged chromosomal version of *PykF*

Gene knockout mutants were created using a linear transformation protocol to introduce a kanamycin cassette into the pyruvate kinase gene [4, 5]. The *pykF*-3×FLAG and *pykF*-*gfp* mutants were created using a two-step PCR reaction. Primers were designed to amplify the kanamycin cassette from the pKD4 plasmid, putting the tag at the 5’ end. The primers also added 40-bp sequences at the 3’ end of *pykF*. For *pykF*-3×FLAG, the primers used were 1F: TTACAAAGATCACGACGGCGATTATAAAGACCATGATATCGATTATAAAGATGACGACGATAAATAAtattgcttGTGTAGGCTGGAGCTGCTTC (where the first part is the FLAG-tag sequence followed by a stop codon, the lower case part is a linker, and the final part is the start of the kanamycin resistance cassette); 1R: AAAGCGCCCATCAGGGCGCTTCGATATACAAATTAATTCACATATGAATATCCTCCTTTAG (where the first part is complementary to the sequence immediately 3’ of *pykF*, and the underlined part is complementary to the end of the kanamycin cassette); 2F: ACCGAGCGGCACTACTAACACCGCATCTGTTCACGTCCTGGATTACAAAGATCACGACGG (where the first part is the 40 bases at the 3’ end of *pykF*, and the underlined part is the start of the FLAG tag); 2R: AAAGCGCCCATCAGGGCGA (identical to the start of 1R). For *pykF-gfp*, the primers used were 1F: ACCGAGCGGCACTACTAACACCGCATCTGTTCACGTCCTGCGTAAAGGAGAAGAACTT (first part is 3’ end of *pykF*, underlined part is 5’ end of *gfp* from GFPmut3.1), 1R: GAAGCAGCTCCAGCCTACACTTATTTGTATAGTTCATCCA (first part is complementary to 5’ end of kanamycin cassette, underlined part is complementary to 3’ end of *gfp*); 2R GCGCCCATCAGGGCGCTTCGATATACAAATTAATTCACAACATATGAATATCCTCCTTAG (first part is complementary to 3’ end of kanamycin cassette, underlined part is complementary to sequence 3’ to *pykF*). PCR reaction products were run out on an agarose gel and purified using a kit (Qiagen).

Competent cells were prepared using *E. coli* pSIM18. The PL promoter in pSIM is controlled by a temperature-sensitive lambda phage cI857 repressor [6]. The competent cells were transformed by electroporation at 1800 V in a Hybaid Cell Shock Electroporator. Transduction with P1vir1 phage was used to move the gene into a new *E. coli* MG1655 background as a stable single-copy insertion [5]. A P1 lysate was prepared by growing donor cells with diluted P1 phage stock in soft-top LB-agar containing 1% glucose (w/v) and 2.5 mM CaCl_2_. This was serially diluted, mixed with recipient cells and plated on LB agar plus kanamycin plus 125 μM sodium pyrophosphate (to bind Ca^2+^ and prevent phage infection). Transductants were checked by DNA sequencing and the absence of P1vir1 confirmed by cross-streaking.

References

1.	Bennett BD, Kimball EH, Gao M, Osterhout R, Van Dien SJ, Rabinowitz JD. 2009 Absolute metabolite concentrations and implied enzyme active site occupancy in *Escherichia coli*. *Nature Chem. Biol.* **5**, 593-599.

2.	Hecht E. 2001 *Optics*, 4th edn. Boston: Addison-Wesley.

3.	Lindon JC, Ferrige AG. 1980 Digitization and data processing in Fourier transform NMR. *Progr. Nuclear Magn. Reson. Spectrosc.* **14**, 27-66.

4.	Datsenko KA, Wanner BL. 2000 One-step inactivation of chromosomal genes in *Escherichia coli* K-12 using PCR products. *Proc. Natl. Acad. Sci. USA* **97**, 6640-6645.

5.	Murphy KC. 1998 Use of bacteriophage λ recombination functions to promote gene replacement in *Escherichia coli*. *J. Bact.* **180**, 2063-2071.

6.	Chan W, Costantino N, Li R, Lee SC, Su Q, Melvin D, Court DL, Liu P. 2007 A recombineering based approach for high-throughput conditional knockout targeting vector construction. *Nucleic Acids Res.* **35**, e64.
