# Supplementary material for: Homeostasis of metabolites in Escherichia coli on transition from anaerobic to aerobic conditions and the transient secretion of pyruvate
Source: R Soc Open Sci. 2016 Aug 24;3(8):160187. doi: 10.1098/rsos.160187 (PMC5108944; doi:10.1098/rsos.160187)
Supplement: files to zip.zip [file rsos160187supp2.zip › files to zip.tar!/table S1.doc]

**Table S1**

**Magnitude of sources of errors for NMR detection of metabolites from chemostat growths**

| Source of error | % error (total)^a^ | % error (individual) ^a^ |
| --- | --- | --- |
| Integration | 0.6 | 0.6 |
| Sampling | 1.8 | 1.2 |
| Biological variation (supernatant) | 2.1 | 0.3 |
| Quenching | 16.0 | 14.2 |
| Biological variation (intracellular) | 20.6 | 4.5 |

Errors were determined by repeated measurements, using different types of repeats to measure different sources of error.

*Integration error* is the error from processing and integrating the NMR raw data, and was estimated by repeated processing of identical FIDs. If the baseline is flat and signals are reasonably sharp, the biggest integration error comes from poor signal-to-noise , and was minimized by acquiring up to 1024 scans for the less sensitive intracellular measurements. Various automated methods were tried for setting integration limits, baseline correction and phasing, but the most reproducible results were obtained by doing these manually. The error reported is the standard deviation for measuring the relative intensity of two signals, expressed as a percentage of the peak intensity.

*Sampling error* is the error arising from independent sampling and handling of the same biological sample, and was determined by collecting several samples from the same culture at the same time and measuring them independently. This includes pipetting errors.

*Biological variation error*, as measured for supernatant (extracellular) samples, is the variation arising from the fact that different biological samples may be different. It was determined by preparing different steady-state cultures, starting from the same starter culture (stored at -80˚ in aliquots). The biological variation error for intracellular samples is likely to be much larger (and indeed is much larger), because one would expect that intracellular concentrations from different cultures vary more than extracellular ones, because of both natural variation and handling. Contamination of intracellular samples by the medium is a much bigger problem than contamination of extracellular samples by cell leakage, because intracellular volume is very small compared to the extracellular volume.

*Quenching error* comes from experimental variability in the quenching process. Because the supernatant volume is so much larger than the intracellular volume (and there is no observable leakage of metabolites), quenching error only affects intracellular measurements.

^a^The *total error* (column 2) was assumed to be the sum of the *individual errors* from the relevant different sources (column 3). Repeated processing and integration of the same dataset generates a total error which arises entirely from individual integration error. Therefore the total error for integration is the same as the individual error. The total error for repeated sampling of the same culture, followed by processing and integration of the results, is the sum of individual integration and sampling errors. The individual error for sampling can therefore be obtained by subtracting the individual integration error from the total sampling error, the difference being assumed to be only due to sampling error. The total error in measuring extracellular supernatants from different cultures is the sum of integration, sampling and (supernatant) biological variation errors. The individual error for extracellular biological variation was therefore obtained by subtracting the total sampling error (or equivalently, the sum of the individual sampling and integration errors). The total error in measuring intracellular metabolites in repeated samples of the same steady-state culture should be the sum of integration, sampling and quenching errors, while the error in measuring intracellular metabolites from different but repeated cultures is the sum of integration, sampling, quenching and biological variation error. The last two individual error values listed in the table are therefore a compromise, since the experimental data do not provide a completely consistent set of error values (implying that some of the errors may not be independent of each other).

1.	Lindon JC, Ferrige AG. 1980 Digitization and data processing in Fourier transform NMR*. Progr. Nuclear Magn. Reson. Spectrosc***.** 14, 27-66.
